# State-of-the-Art of Analytical Techniques to Determine Food Fraud in Olive Oils

**DOI:** 10.3390/foods10030484

**Published:** 2021-02-24

**Authors:** Antia González-Pereira, Paz Otero, Maria Fraga-Corral, Paula Garcia-Oliveira, Maria Carpena, Miguel A. Prieto, Jesus Simal-Gandara

**Affiliations:** 1 Nutrition and Bromatology Group, Analytical and Food Chemistry Department, Faculty of Food Science and Technology, Ourense Campus, University of Vigo, E-32004 Ourense, Spain; antia.gonzalez.pereira@uvigo.es (A.G.-P.); pazoterofuertes@gmail.com (P.O.); mfraga@uvigo.es (M.F.-C.); paula.garcia.oliveira@uvigo.es (P.G.-O.); maria.carpena.rodriguez@uvigo.es (M.C.); 2 Centro de Investigação de Montanha (CIMO), Campus de Santa Apolonia, Instituto Politécnico de Bragança, 5300-253 Bragança, Portugal

**Keywords:** food fraud, optimum analytical methodology, techniques of spectroscopy and spectrometry, chromatographic methods, recent analytical approaches

## Abstract

The benefits of the food industry compared to other sectors are much lower, which is why producers are tempted to commit fraud. Although it is a bad practice committed with a wide variety of foods, it is worth noting the case of olive oil because it is a product of great value and with a high percentage of fraud. It is for all these reasons that the authenticity of olive oil has become a major problem for producers, consumers, and legislators. To avoid such fraud, it is necessary to develop analytical techniques to detect them. In this review, we performed a complete analysis about the available instrumentation used in olive fraud which comprised spectroscopic and spectrometric methodology and analyte separation techniques such as liquid chromatography and gas chromatography. Additionally, other methodology including protein-based biomolecular techniques and analytical approaches like metabolomic, hhyperspectral imaging and chemometrics are discussed.

## 1. Introduction

Food industry has narrow profit margins compared to other sectors. In order to increase those profits, unethical sellers try to maximize incomes through counterfeiting and adulteration practices, a procedure known as food fraud (FF). FF is a collective term used to encompass the deliberate and intentional substitution, addition, tampering, or misrepresentation of food, food ingredients, or food packaging; or false or misleading statements made about a product, for economic gain [1].

**Figure 1:**
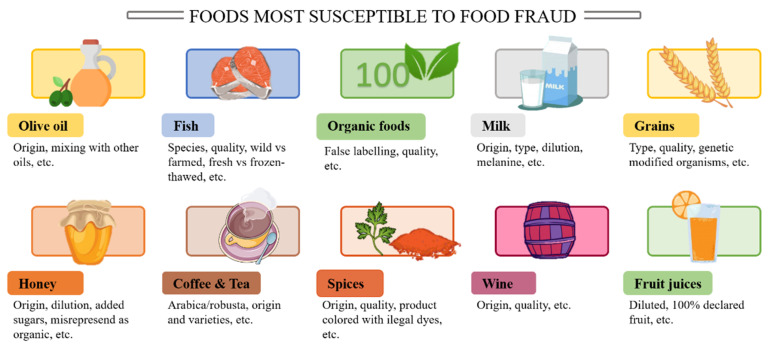
Some of the foods most susceptible to food fraud.

While there is no actual data on the volume of fraud that exists, the costs of global FF is estimated in $ 10–15 billion, being affected about 10% of all foods sold [2]. It is expected to increase in the next years, due to the increasing vulnerability of the supply chain, due to its lengthening and the globalization [1]. FF affects to several products including beer, spirits, fish, grains, olive oil, organic foods and may others (Figure 1). From them, oils fraud and especially olive oil fraud is of extreme importance due to the difference in the quality, properties and price between authentic and adulterated oil. According with the 2019 Annual Report of the European Union Food Fraud Network, the category ‘Fats and oils’ was the one with the highest number of requests, olive oil being the most notified of the system [3]. This supposes, for example, that about 80% of the Italian extra virgin olive oil on the market is fraudulent. Within this percentage, most of the fraud committed is due to the addition of economical vegetable oils (palm oil, palm stearin olein, etc.), whether refined or processed [4]. The refinement of olive oil is not an interesting process because it loses their properties. The fact that olive oil can be consume without extensive refining makes the possible fraud be considered of high relevance. Two different kinds of adulterations are possible: the addition of lower quality oils from the same specie (refined olive oil or olive pomace oil) and additions of oil from other species [5]. It should be highlighted that oil is a complex matrix containing triacylglycerols, partial glycerides, hydrocarbons, tocopherols, pigments, sterols, alcohols, triterpene acids, volatile compounds, phenolic compounds, phospholipids, and proteins [6]. The lipid composition is characteristic of each specie, and therefore it is a suitable indicator of adulteration. For example, (E)-5-methylhept-2-en-4-one (filbertone) is present in hazelnut oil [7], brassicasterol in canola oil and sesamol, sesamin, or sesamolin in sesame oil [8]. Consequently, to perceive edible oils and fats adulteration, it is possible to use both major and minor compounds as detection tool since each oil may have an especial component at a known level [9]. Some extra virgin olive oils (EVOOs) have been reported to present high quality characteristic depending on the cultivar or region, so FF may also occur if the product is not from the declared country or region [10].

**Figure 2:**
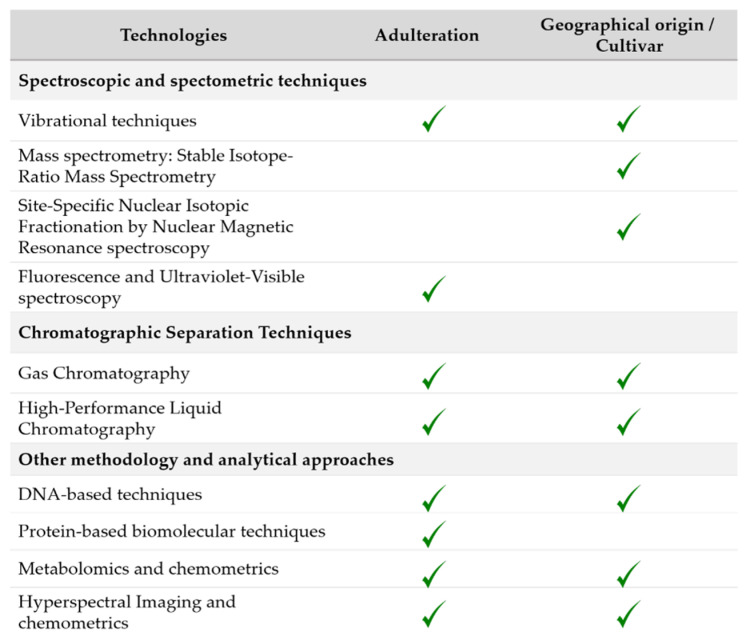
Applications of the selected techniques for olive oil authentication.

A few decades ago, physical parameters such as refractive index, viscosity, melting point, saponification, and iodine value were used to detect these FF. Nowadays, they are obsolete techniques, since adulteration process has advanced and has become more complex [9]. These continuous advances make the development of detection techniques a real challenge. Even so, analytical techniques allow detecting most of them [11]. Nowadays, the European Union Commission, International Olive Council and Codex Committee on Fats and Oils are working in the regulation and control of EVOO quality. These organisms have specified quite similar permissible limits for EVOO quality parameters and have also established the official methods for quality control and the detection of possible fraud. Regarding European Union Commission, Regulation (EU) 1348/2013 and Regulation (EU) 2015/1833 amending Regulation (EEC) No 2568/91 establish the characteristics of each olive oil type and include several relevant analytical methods, most of them based on chromatographic techniques. However, some of the recommended methods present drawbacks, such as complexity, excessive use of toxic compounds, laborious sample preparation, etc. [12]. Thus, numerous advances and other analytical techniques have been developed to overcome these problems, being useful to detect oil adulteration and fraud. The aim of this review is to present the most significant techniques and examples of their application in oil authentication (Figure 2).

## 2. Spectroscopic and Spectrometric Techniques

Among the spectroscopic (SP) techniques, infrared, near-infrared, mid-infrared, nuclear magnetic resonance, and ultraviolet-visible spectroscopy are widely used in food authentication [13]. Some studies carried out with these techniques can be observed in Table 1. In general, it has been described that these techniques present several advantages, like low running cost, rapidity, they are non-destructive and no or minimum sample preparation is needed [12,14,15]. In the following paragraph, the basis of each technique and also examples of their use for the determination of EVOO adulteration will be explained.

### 2.1. Vibrational Spectroscopy

#### 2.1.1. Fourier Transform Infrared Spectroscopy

Fourier Transform Infrared Spectroscopy (FT-IR) constitutes a broadly technologically advanced vibrational spectroscopy instrumentation employed to determinate molecular structure of organic samples. The principle of this technique is based on the energy absorbed by the functional groups’ linkages (hydroxyl, carboxyl, *nitrogen* hydrides, etc.) and the emitted vibrations when compounds are under electromagnetic radiation (for example light), being the vibratory mode characteristic of each molecular group [38]. This method is fast and non-destructive, it requires the minimal sample preparation and permits the qualitative determination of molecules, based in their vibratory mode. For all these characteristics, this technique is considered an emerging analytical procedure for the validation of the edible oils and fats genuineness [39]. In fact, a study comparing this technique with others SP methods (Raman and NIR) proved that FT-IR provided high precision and achieved the best results in classifying oils, with accuracy yields of approximately 98%. FT-Raman and FT-NIR displayed accuracy levels of 94% and 93%, respectively [39]. Nowadays, FT-IR technique is applied for the adulteration of EVOO with lower priced vegetable oils. For example, Tay and co-workers [40] tested the method effectiveness by analysing pure olive oil with different quantities of sunflower oil in the range of 0.02–0.1 L per L olive oil, showing a successful discrimination among them [40]. Another study also applied FT-IR to distinguish unequivocally different oil types (palm, corn, canola and sunflower) and to detect EVOO adulterated with palm oil in quantities up to 50% (*w/w*) [25]. Finally, this technique was also applied to hazelnut oil adulterations in sunflower and olive oils and it was able to detect hazelnut additions in a 2% and 25%, respectively [24]. Although most studies focus on EVOO, sunflower, corn, soybean and hazelnut since they are the most common species, there have also been cases of adulteration by tea seed oil. This fraud can be detected by analysing frequency regions of 4000–650 cm^−1^ [26]. Therefore, this technique allows to distinguish the botanical origin of the sample. In addition, the method was applied to adulterations in dietary supplement oils (DSO). Ozen and co-workers analysed 14 types of DSO adulterated up to 20% (*v/v*) with successful results, with a detection limit of 2% (*v/v*). Hence, FT-IR together with chemometric analyses are efficient techniques to classify oils types from dietary supplements [41].

#### 2.1.2. Fourier Transform Near-Infrared Spectroscopy

The use of Fourier Transform Near-Infrared spectroscopy (FT-NIR) in food quality is based on a spectroscopic fingerprint of each food, associated to the occurrence of a spectra typical range, without considering variations between batches, variety, season or locations [42]. The existence of libraries of representative food spectra allows to compare them with the unknown food and thus, to establish its authenticity [43]. FT-NIR has several advantages, such as quickness, simplicity, is non-destructive, simple (no sample pre-treatment is needed), and the equipment calibration. Moreover, it requires a small solvent volume, gives the possibility of measuring various compounds at the same time and is suitable for remote applications [44,45,46]. Against the previous FT-IR, in which only specific functional groups can be quantified, FT-NIR permits the complete fatty acid profile elucidation of an oil in few minutes without sample derivatization, unlike GC official methods [47]. However, the technique has some drawbacks. For example, NIR spectrum may contain interferences from noise and overlapping bands [14], and the low signal sensitivity make the detection of low compound concentrations difficult [48].

FT-NIR was applied for the first time in 1991, when it was developed in a new oil spectra library to elucidate the origin of unknown samples [49]. Since then, the use of this technique has increased, especially in quality control processes during the edible oil production. It allows to verify important parameters (moisture, free fatty acids composition), to evaluate bacteria, fungi, and mold growth, and increase extraction efficiency [50]. In addition, the methodology is used to evaluate transformation processes like fractionation and hydrogenation, as well as the physiochemical parameters of final products and oil by-products intended for animal feed [47]. Additionally, it is worth mentioning that this technique has high potential to quickly classify and quantify the saturated, monounsaturated and polyunsaturated fatty acids, allowing comparisons of unknown fatty acids and oils qualitatively and quantitatively [51].

Bibliography described numerous examples of FT-NIR applications. For example, it can be used for sunflower oil detection in EVOO with extremely high precision in the range of 1100–2498 nm (standard error of 0.8%) [19]. FT-NIR is also able to detect soybean oil in EVOO with a R2 greater than 0.98 [16]. This technique is also effective in determining low quality oils (corn, sunflower, soya, walnut, and hazelnut oil) in EVOO by analyzing the regions of 12,000–4000 cm^−1^ [17]. In this case, models showed adulterations of several oils with error limits of ±0.57 (corn), ±1.32 (sunflower), ±0.96 (soya), ±0.56 (walnut), and ±0.57% (hazelnut) (*w/w*), respectively. Other models accurately determined adulterated olive oil mixtures [18]. Similar results were obtained in olive oil adulterated with sunflower and corn oil in the range of 4–96% (*v/v*). The standard errors ranged from 2.49 to 2.88% (*v/v*) in the mixtures of olive and sunflower oil and from 1.42 to 6.38% (*v/v*) in the mixtures of three oils (olive, sunflower, and corn) [52]. Therefore, FT-NIR can detect the botanical origin of the samples and discriminate between types of edible oil, detecting even low quantities of adulteration in EVOO [53].

Moreover, this technique allows to detect the adulteration content of the EVOO in a rapid process by applying different chemometric algorithms. Among them, bootstrapping soft shrinkage showed superiority in the selection of informative wave numbers [53]. Another one is partial least squares (PLS) algorithm, which discriminated and quantified 280 samples of olive oil adulterated with corn, hazelnut, soybean and sunflower oils in the regions 12,000–4550 cm^−1^ [54]. PLS has also been applied to distinguish among four oil formulations and determine the iodine number, proving that FT-NIR in combination with PLS can identified oil type and iodine number with a high degree of confidence, which allows an improved control of the process to which the oils are subjected [55]. This technique allows to evaluate simultaneously the content of cis and trans fats, the iodine number and the saponification number of pure fats and oils with a precision and reproducibility of the order of ±1.5 and ±1.0 units for all evaluated parameters [56]. This combination of methods can be applied in other wavelengths with good results. For example, Azizian et al. [20] analyzed volatiles compounds at 5280 cm^−1^ and non-volatile components at 5180 cm^−1^ for predicting fatty acid composition of EVOO and samples enriched with an adulterant. As a conclusion, some adulterated blends could be identified if the fatty acid profile was sufficiently different from that of EVOO [20]. Moreover, FT-NIR spectroscopy constitutes a robust alternative to the SB-HATR/mid-Fourier transform infrared method for trans determination in the industrial processes [57].

#### 2.1.3. Raman Spectroscopy

Raman spectroscopy is based on the detection of molecular vibrations in a sample, induced by an incident light source. The interactions between the sample molecules and photos depend on the properties of the sample and the wavelength of the light (visible, infrared or UV) [58]. Raman spectroscopy is a non-invasive and non-destructive technique, its methodology is simple, and no sample pre-treatment is needed. Moreover, the portable Raman system is suitable for on-site testing [31]. However, it provides low signals, overlapped peaks and low operational speed. Other disadvantages are the interferences by strong fluorescence backgrounds and spherical aberration or refraction [59]. Oils and fats are the most common food studied with FT-Raman spectroscopy due to their phase homogeneity and non-polar chemical nature, therefore it is a promising tool to detect authentication and adulteration of olive oil [60]. In general, to evaluate oil samples, the wavelength excitation of the laser is usually in the visible or infrared range [61,62,63].

Different research works have been performed in this regard. Samples of olive oil were altered with soybean, corn, and sunflower seed oils, and then it was possible to corroborate it analysing the region of 1000–1800 cm^−1^, which reveals that Raman is a promising technique for the authentication of extra virgin olive oil [31]. In another study, Raman spectroscopy was employed to quantify soybean oil adulteration [up to 25% (*w/w*)]. A PLS Regression model was validated (in the region 1000–1800 cm^−1^), achieving high correlation coefficient of prediction [64]. It was also useful for distinguishing between closely related extra virgin olive and hazelnut oils. [65].

Another common way to adulterate the oil is by adding waste cooking oil and again the use of this technique revealed to be a proper tool to figure it out. The result of a study analysing 96 samples of olive oil with used cooking oil (2.5%, 5%, 10%, 20%, 30%, and 50%) and for which quantitative analysis models were established IPLS and SiPLS, revealed that spectral data after SNV processing is the best for modelling and predicting partial least squares synergy intervals (SiPLS) [32].

Moreover, this technique also allows obtaining information on the unsaturation degree. The scattering intensities near different Raman shifts (3013, 1663, and 1264 cm^−1^) show high correlations with the fatty acid profile determined by gas chromatography. For this purpose, different vegetable oils and some mixtures were employed as calibration standards. A calibration model based on PLS was constructed and used to analyse oils with iodine values ranging from 17 to 130 [66]. Recently, Raman spectroscopy has been employed to discriminate the type of cultivar [67,68] and the geographical origin of EVOO samples [62], achieving good classification results.

### 2.2. Mass Spectrometry: Stable Isotope-Ratio Mass Spectrometry (IRMS)

Mass spectrometry (MS) is an analytical tool that permits to study the chemical composition of a sample by the analysis of the mass-to-charge ratio (*m/z*) of produced ions. This technique allows both quantitative and qualitative approaches [38]. MS can be utilized as a single analytical instrumentation or it can be coupled to chromatographic or even spectroscopic instruments to provide more complete and defined results. In the field of the authentication, the measurement of natural isotopic abundances has been showed as a useful technique for the identification of adulterations. The instrument used for the study of stable isotope ratio is a multi-collector magnetic sector mass spectrometer, also known as IRMS [69].

IRMS can discriminate the abundance of some isotopes ^13^C/^12^C, ^15^N/^14^N and ^18^O/^16^O by measuring their atomic masses. Obtained values permit the differentiation of samples based on their isotopic footprint. This is a unique pattern of isotopic values that allows the determination of the geographical origin and the authentication of food and beverage samples. Sample pre-treatment for IRMS is time-saving and simple. Before samples get into IRMS they must be converted to simple gases such as N_2_, H_2_, CO_2_, or CO. Among the interfaces coupled to IRMS, the most used is the elemental analyzer (EA). Besides, IRMS can be also coupled to chromatography instruments such as liquid chromatography (LC) or gas chromatography (GC). The advantages of using this method includes its high sensitivity and precision. However, when testing unknown samples, it is crucial to use well characterized standards with isotope ratios established against international standards [70] (Equation (1)).

(1) $$\delta =\frac{1000\;\left(Rsample-Rstandard\right)}{Rstandard}$$

Different approaches of the technique have been applied to provide geographical, temporal, and botanical authentication criteria for very different samples, among which are oils, especially those obtained from olives. Indeed, olive oils have been widely analyzed using this method. For instance, a study based on the ratio ^13^C/^12^C (δ^13^C) of fatty acids from olive oil samples from different origins (France, Italy, and Greece) was able to differentiate them. Using the GC-^13^C-IRMS the isotopic parameters of whole oils and commercial fatty acid methyl esters were determined. According to the results of this study, the method was able to differentiate oleic (C18:1) from linoleic (C18:2) acids and oleic from palmitic (C16:0) acids. However, no significant differences were found between linoleic and palmitic acids. The results showed that the region of production provided different δ^13^C values, being oils from France and Italy those that were better identified [71]. Another two studies characterized chemically and isotopically the fatty acids of olive oils from different origins and quality, including thermal induce degradation analysis. Both studies verified the substantial enrichment in the heavy carbon isotope (^13^C) of both bulk oil and fatty acids when thermal degradation was induced. Thermal degradation may be due to deodorization or steam washing of olive oils, but other factors may be involved in this enrichment, such as the blend with refined oils, lipolysis, and/or lipid oxidation triggered by long term storage [72,73]. IRMS was also used to analyze δ^13^C and δ^18^O of EVOO from selected areas of Italy. Samples from hot climate regions such as Sicily showed relatively high δ^18^O and δ^13^C values. However, sample clustering is not definitive and did not allow the identification of subareas by itself. Nevertheless, this study found that data can be corrected when complemented with results from other analysis such as Raman spectroscopy or carotenoid content [62]. Same approach, evaluation of δ^13^C and δ^18^O by IRMS, was used to analyze cleavage products (free glycerol and fatty alcohols) from food fats of different origin. Glycerol from olive oils from defined origins and glycerol from commercial samples with diverse geographical origins were analyzed to obtain authenticity parameters. The established method was able of differentiating natural from synthetic glycerol based in δ^13^C and δ^18^O values. However, for fatty alcohols just the δ^13^C value was useful for determining its authenticity [74]. Extra virgin olive oils from Portugal and Turkey were also used to evaluate the isotope ratios of three of their fatty acid methyl esters which showed differences related with their geographical origin [75]. Similarly, stable isotope ratios (δ^13^C, δ^2^H, δ^18^O) of bulk olive oils and EVOOs obtained from over-world countries were evaluated using IRMS as a tool for determining its geographical origin. Moreover, δ^13^C and δ^2^H values of olive oils were identified with GC-IRMS for linoleic, oleic, palmitic and stearic acid. The isotopic fingerprint of tested oils was not directly related to individual countries but to climatic, geographical and geological characteristics. In fact, other paper about Portuguese oils reached same results being able of predicting altitude, latitude, longitude, temperature, rainfall, and sea distance using same methodological approach [76,77].

Another kind of oil, rapeseed oil, has been also evaluated with IRMS based techniques. Stable isotopes, δ^13^C, δ^2^H, δ^18^O, of bulk oils and δ^13^C of individual fatty acids were analyzed. Additionally, other vegetable oils rich in linolenic (flax oils) and linoleic acids (poppy, sunflower, and safflower oils) were identically determined. The δ^13^C value of individual fatty acids provides differences between species. Specifically, rapeseed, flax, and poppy oils were differentiated by the δ^13^C value of the palmitic and n-3 α-linolenic acids. Whereas diverse cultivars of rapeseed oils were identified through δ^2^H and δ^18^O values [78]. The same technique, GC-C-IRMS, using the δ^13^C measurement was used to authenticate another kind of oil: bergamot essential oil. Analysis of the δ^13^C value for several of the major compounds of the essential oil, such as pinene, limonene, linalool, among many others, were evaluated to use as quality control. Those were compared against bergamot essential oils obtained from market, from other regions, and intentionally adulterated samples. Results determined deviations in samples from other regions (both commercial and from Ivory) and adulterated, being even capable of discriminating the nature of the adulterants added [79]. Therefore, the analysis of stable isotopes of oil in bulk and specific fatty acids represents an useful tool to track diet lipids with different origins [78].

### 2.3. Site-Specific Nuclear Isotopic Fractionation by Nuclear Magnetic Resonance (SNIF-NMR) Spectroscopy

Nuclear Magnetic Resonance (NMR) spectroscopy is based on the application a magnetic field (4–900 MHz) to atomic nuclei that possess magnetic properties. Thus, nuclei with odd atomic or mass number or both, like ^1^H, ^13^C, ^15^N, ^17^O, ^19^F, ^23^Na, ^29^Si, or ^39^K, among others, provide very useful information in NMR. Nuclei when exposed to a magnetic field at an appropriate radio-frequency radiation can absorb energy. This energy is further transformed, recorded as resonance signal and encoded to produce spatial information and finally provide NMR images [80]. Nowadays, NMR is widely applied for performing non-targeted analysis of food for its authentication. There are few properties that make it a useful tool in food science such as its unique quantitative properties, excellent linearity, an incremented proportionality of response-concentration, adjustable sensitivity and low detection limits [11]. ^1^H NMR spectroscopy has been widely applied for characterizing cultivars and geographical origin of EVOO and other edible oils [81,82]. Among the multiple variants of this technique, the site-specific natural isotopic fractionation studied by NMR (SNIF-NMR), developed in the 80s by Gerard Martin and Maryvonne Martin, has been specifically and repeatedly utilized in food science since it allows the determination of the geographical and chemical origin of a molecule [83].

Many natural processes are accompanied by the isotopic fractionation of atoms. Hence, the determination of the isotopic abundance has become a useful tool to determine the natural or synthetic nature of molecules [84]. SNIF-NMR can accurately quantify different hydrogen isotope ratios in each position of a molecule. Different factors such as botanical origin, climate and geography affect this pattern, resulting into an isotopic fingerprint, which ultimately provides a tool for determining the authenticity and/or the origin of the product itself. In fact, SNIF-NMR has been chosen as the official method of analysis for a variety of European organizations such as the International Organization of Vine and Wine (OIV), the Association of Analytical Chemists (AOAC), and European Committee for Standardization (CEN) [84,85]. The main drawbacks of this authentication tool are its relatively low sensitivity, its requirement of an impurity profile and analysis may become time-consuming. On the other hand, the main advantages include that it does not require the application of time-consuming sample pretreatments, such as concentration and purification steps, and that it gives intramolecular δ^13^C information [86]. In the last three decades, few methodological developments have updated this technique, among them is worth to underline its extension to ^13^C isotopic and anisotropic ^2^H NMR, which has allowed the expansion of its application including the inclusion other molecular targets apart from sugars [84]. The use of the last modality, ^2^H NMR, has been especially suitable to characterize nearly two hundred olive oil samples in terms of botanical and geographical factors. Samples included different factors to evaluate. They were selected different oil classes, including EVOO, from four countries (Greece, Italy, Spain, and France), different temporal productions, belonging to several botanical varieties and collected at diverse ripening stages. Differences in the isotope ratio showed differences between the oil classes, besides the ^2^H spectra of lipids from olive oil were demonstrated to be influenced by climatic variations. The ^2^H and ^13^C distribution is variable according with the region and the production period, while other factors like the variety and ripening degree are not so relevant [87].

### 2.4. Fluorescence and Ultraviolet-Visible (UV-Vis) Spectroscopy

UV-visible spectroscopy is based on the UV or visible light absorption by chemical compounds producing different spectra [88]. The importance of this technique lies in its high sensitivity and excellent specificity. In fact, spectrofluorometric methods can detect components with a sensitivity of 1000 times higher than other spectrophotometric techniques [89]. However, some of the major drawbacks are the strong dependence on light scattering and the lack of mathematical corrections because the spectrum does not contain information on the amount of scattering. Furthermore, it is highly dependent on environmental conditions (pH, ionic strength, viscosity, or temperature) which must be controlled to obtain reproducible measurements [89].

This technique is applied to the analysis of the changes produced in virgin olive oil during storage since the intensities of pigments and tocopherols normally decreased during the storage. It is possible to observe bands attributed to tocopherols and chlorophylls which allows to monitor the effects of storage on these compounds [90,91]. It also serves to detect frying oil additions in the range of 1% to 25% [34]. To determine adulterations in olive oil, a model of spectra ranging from 400 to 550 nm was developed. It allows to compare the unknown samples with unadulterated samples officially categorized as EVOO. For instance, to detect adulterations of argan oil in olive oil, the spectra at 532 nm is analysed and the detection of the sensitivity of adulteration is possible from 0.43% olive oil mixed with 99.57% argan oil (*w/w*) [92]. Instead, to analyse adulterations of the extra virgin olive oil with other type olive oil, the region of interest is between 60 and 700 nm. In this case the lowest adulteration detection limits were 8.9% and 8.4% when the wavelength interval applied was 60 and 80 nm, respectively [35].

## 3. Chromatographic Separation Techniques

### 3.1. Gas Chromatography (GC)

This technique is based on the study of compounds in a gaseous state, so the analytes of interest must easily vaporize without decomposing. The application of this technique is especially useful to analyze aromatic compounds, mainly in combination with mass spectrometric detection. GC-MS coupling is the most widely used technique (>50%), followed by GC coupled to other types of detectors [93]. This technique has the advantage of requiring a small amount of sample and detecting compounds at very low concentrations. On the contrary, most of the molecules are neither volatile nor thermolabile and cannot be analyzed by GC. Thus, many studies have focused on developing derivatization methods that increase analyte volatility [94].

This technique applied to the authentication of edible oils and fats has achieved great results. As mentioned before, these foods are mainly made up of saturated and unsaturated fatty acids (from C12 to C22) esterified with glycerol-forming triacylglycerols, and small amounts of sterols, terpenic alcohols, hydrocarbons, vitamins, etc. This technique is able to detect qualitative and quantitative differences in the mentioned compounds that allow to differentiate the oils, due to the different biosynthetic pathways characteristic of each species [95]. The analysis of triglycerides has made it possible to detect fraudulent additions of seed oils to olive oil and adulterations in different fats [96].

Another study focused on analyzing individual species of olive oil triglycerides and various seed oils (corn, cottonseed, palm, peanut, soybean, and sunflower) for the determination of adulterations. Low contents (<5%) of these seed oils (except peanut oil) were detected in olive oil due to the detection of increasing levels of trilinolein or tripalmitin. In the case of peanut oil, adulterations of more than 20% in olive oil can be detected due to increasing levels of palmitodilinolein. However, it was not possible to detect the addition of refined olive oil by the method applied in the same study [97].

Therefore, GC allows to distinguish pure oils from mixtures and to discriminate between different types of seed oils used for adulteration. This is possible not only by determining their composition, but also by determining the molar percentage of total fatty acids and their regiospecific distribution in positions 1 and 3 in triglycerides of oils (pure or mixtures) by GC analysis [98]. This technique has been improved by using stationary carborane-based columns that can reach temperatures of up to 480 °C [99]. In this way, additions of 5% of the different vegetable oils to olive oil can be detected based on the study of the presence of campesterol and the content of stigmasterol [100]. Another widely used column is HP-5 (5% phenyl; 95% dimethylpolysiloxane), which is used to identify adulterations of Chemlali extra-virgin olive oils with sunflower oil (by the increase of Δ7-stigmastenol) and with corn oil (by the increase of campesterol) [101] (Table 2).

This technique is also useful to determinate the olive variety and the origin employed to obtain the oil. For example, the combination of GC coupled to flame ionization detector and multivariate classification techniques allowed to differentiate three EVOOs from Arbequina cultivar according to their geographical region [102]. Another study proved that GC-MS was suitable to verify the geographical origin of Italian EVOOs [103].

### 3.2. High-Performance Liquid Chromatography (HPLC)

HPLC is one of the most versatile analytical techniques that allows the analysis of both polar and apolar compounds and it is widely used in food authentication. This technique consists of separating the compounds of interest between two phases, one of which is stationary while the other is mobile and is made up of a liquid that moves in a defined direction. This instrumental technique is widely used due to its versatility, high sensitivity, easy adaptability, precision, the possibility of using non-volatile or unstable thermal species, and its great applicability to identify and quantify substances of interest in industry or research [115].

Many studies focus on the application of this technique to the detection of adulterations in oils. This technique allows to detect additions of only 1% of vegetal oils rich in linoleic (soybean, sunflower, corn) to olive oil, using a stationary phase of silica linked to octyl (Supelcosil-LC 8) and a mobile phase was acetone-acetonitrile (70: 30, *v/v*) in isocratic regime [116]. Another way to determine adulterations in olive oil is to carry out a triacylglyceride analysis. To do this, the HPLC technique can be applied, obtaining the best resolution using propionitrile at 20 °C. Although the HPLC profile was similar using propionitrile and acetone/acetonitrile, differences were found in the minor triacylglycerols contributing to each HPLC peak. The precision of the method was good [117].

Furthermore, the triacylglyceride analysis is the foundation of the official methods for detecting adulteration of olive oil. For this, the carbon equivalent number is studied (ECN, ECN 42, 44, and 46). This method has been recently adopted by the International Olive Council as the official method for determining the authenticity of olive oils [118].

On the contrary, the sweetening of olive oil with hazelnut oil can only be detected at high proportions (20–25%) using the Δ7-stigmastenol and the difference between carbon equivalent triacylglycerols number 42 as indicator. To avoid this problem, a method that use algorithms was used. The algorithms are based on a database built with data obtained from genuine virgin olive oils, finally achieving detection of low percentages of hazelnut oil in olive oil (5%) [119].

Regarding minor components, tocopherols, carotenoids, chlorophylls, and (phyto)sterols can be also used to detect adulteration. A study focused on the quantification of tocopherols, carotenoids, and chlorophylls in vegetable oil by applying C30 RP-HPLC with electrochemical detection for its simultaneous analysis, obtaining detection limits of 1 fmol, 0.15 pmol, and 0.5 pmol for carotenoids, tocopherols and chlorophylls respectively, being able to apply this method for a rapid and sensitive analysis in the study of the quality and adulteration of the oil. The concentrations of total β-carotene and α-carotene together with the ratio of trans to cis-isomers of β-carotene are reliable indices for fast screening of oils [120].

Another alternative is the application of luminescent methods in the analysis of edible oils without any previous treatment, such as extraction before analysis. This makes it possible to determine quality parameters of edible oils, such as oxidative stability, antioxidant activity and the content of lipid hydroperoxides, as well as the classification or adulteration of vegetable oils. In this way, the authenticity of virgin olive oil based on the concentration of α, ß, and γ-tocopherols has been analyzed by HPLC with fluorescence detection, being able to detect percentages as low as 1.5% and 3% of peanut and hazelnut oils in virgin olive oil, respectively [121].

The analysis of this fraction has also been determined by coupling HPLC with other detectors. The optimization of the interface performance in the on-line coupling of reversed phase liquid chromatography and gas chromatography was intended to improve the sensitivity achievable in the direct analysis of olive oils adulterated with virgin and refined hazelnut oils. The efficient elimination of the eluent coming from the pre-separation was achieved by considering some experimental variables (i.e., transfer volume, interface temperature during transfer, helium flow during both transfer and purge, and purge time) affecting the operation of a vertically positioned programmed temperature vaporizer which acted as the interface of the system. The obtained results demonstrated the possibility of evaluating the genuineness of olive and hazelnut oils as well as of detecting adulterations of olive oil with percentages of around 5% and 10% of virgin and refined hazelnut oils, respectively, in less than 30 min by the method proposed [122].

Polar component analysis can also be carried out. The method, which is based on SPE-based isolation of the polar fraction followed by RP-HPLC analysis with UV detection, can detect virgin olive oil adulterated with pressed hazelnut oil at levels as low as 5% with great results (precision, repeatability, linearity). However, the large variability in marker components among the pressed hazelnut oils examined precludes the use of the method to quantify the level of adulteration [105].

## 4. Other Methodology and Analytical Approaches

### 4.1. DNA-Based Techniques

DNA analysis are based on the evaluation of the genome of the samples, being useful to assess the presence of oils from other vegetal species and also the varietal origin of the product [123]. These techniques are considered highly specific, sensitive, and precise, but they are expensive, thus its routine use is limited. Several studies using DNA-based techniques have shown good results in olive oil authentication. For example, EVOO adulteration with different vegetable oils (maize, sunflower, and hazelnut oils) was assessed by using real-time PCR along with high resolution melting analysis, comparing the DNA melting profiles [124]. In other study, the performance of a DNA barcode assay was compared with GC fatty acid analysis. The results showed that both techniques were equally efficient to detected adulteration of EVOO with other vegetal oils, except in the case of hazelnut, corn and sunflower oils, being the DNA analysis more efficient to detect these adulterations. Regarding cultivar identification, different DNA markers have successfully been employed for this purpose, which have been reviewed previously [125]. These techniques have been proposed as tools for the confirmation of protected designation of origin and protected geographical indication EVOOs [126].

### 4.2. Protein-Based Biomolecular Techniques

Enzymes and antibodies are the most common proteins used for the development of authentication studies. Both molecules can be used as part of the pretreatment. Enzymes can be used to release compounds of interest while antibodies are commonly used to purify a sample. Moreover, enzymes and antibodies can be utilized for performing colorimetric assays. The presence of active/inactive enzymes in the sample permits to monitor the efficiency of food safety protocols such as heat treatment. In a similar way, in the field of oils, the refractive index of the olive oils when treated with different enzymes shows slight changes. Besides, enzymes can be used to transform undetectable products into detectable ones. In the case of antibodies, they can be added into a sample to detect an antigen which will target with a detectable molecule. These assays permit the spectrophotometric determination of many food components, such as organic acids, sugars, or amino acids among others, that will provide a semi-quantitative result [127].

Among the immunoassays, the enzyme-linked immunosorbent assay (ELISA) is one of the most used and known. Enzyme immunoassays are based on the ability of antibodies to recognize antigens. These antibodies can be used to recognize the molecule to analyze or to increase the signal in which case are coupled to an enzyme that catalyze the product transformation into a photodetectable one. This technique has been used in a wide range of applications in food analysis and bioanalytical science [128]. The application of this technique to oils presents disadvantages since the extraction protocol of lipidic samples usually requires a high percentage of organic solvents while because of the biological nature of the antibodies, immunoassays just tolerate small amounts of organic solvent. By other hand, as results from immunoassays are considered semi-quantitative, it is necessary to validate them by comparison with analytical techniques, chromatographic ones in most cases. Thus, the same extraction procedure must be applied otherwise the extraction protocol has to be also validated [129]. All these drawbacks have limited the development of this technique with oil authentication aims. However, few immunoassays have been developed to trace residual proteins in lipophilic matrixes.

An example of its application is the detection of fraudulent additions of hazelnut oil to olive oil. This adulteration represents not just an economic fraud but a public health threat, since it can cause allergy episodes induced by the hinder presence of hazelnut proteins. A highly specific immunoassay for detecting this fraud was developed. The test was based on the use of a monoclonal antibody that provides accurate results due to the high specificity and low sensibility (detection limit of 80 ng/g of hazelnut proteins in olive oil) [112].

Another kind of immunoassay is that based on an immunochromatography. In this case, the antibody is utilized to retain specific compounds that elute along a chromatographic system. Immunochromatography has been employed for the detection of the micotoxin, aflatoxin B1. The presence of micotoxins in products destined for consumption is considered an important food safety issue since their bioaccumulation represents a threat for human and animal health. The application of monoclonal antibodies for the development of immunochromatography assays for detecting aflatoxin B1 offers an economic and rapid test. Working with monoclonal antibodies improves selectivity and avoids false positives that other antibodies can rise caused interference due to the coexistence of other aflatoxins [113]. Other dangerous molecules that can be cheaply and quickly detected with immunoassays are pesticides. An immunosensor was used based on a photovoltaic sensor that transforms fluorescent radiation into electrical signals. Antibodies marked with fluorophores are used to estimate the number of chemical residues from pesticide treatments that are present in olive oil samples [114]. Another danger molecule, diisobutyl phthalate, an endocrine disruptor can be released from packages and it has been found in oils. Its presence can be determined by a very sensitive fluorescence immunoassay which can reach a very low detection limit (5.8 ng/mL in buffer) in optimal experimental conditions [130].

Even though immunoassays account for many drawbacks, its convenience to detect proteins in fatty matrixes is still under discussion since these assays provide a very simple and cheap solution for analyzing samples which requires no trained personnel, economic laboratory reagents and devices, and offers very quick results.

### 4.3. Metabolomics and Chemometrics

Metabolomics is a discipline that identifies and quantifies numerous low molecular compounds (metabolites). The most general aim of this noninvasive technique is to understand the mechanism of action of metabolites belonging to a biological system. However, this tool has been adapted to evaluate the metabolomic profile and to establish chemical fingerprint of specific samples. Thus, ultimately it allows the identification and quantification of adulteration processes and even recognizing the geographical origin of those samples [131]. Among the metabolites used with FF purposes in edible oils, sterol profile has been pointed out as useful markers to determine adulteration or authentication [131,132]. Several techniques, such as NMR, MS, and few based in vibrational spectroscopy, have been developed to study the metabolome of biological samples. The application of more than one technique provides a more complex scenario that brings closer to the huge complexity of the metabolome [38]. The compilation of such complex matrix of data and experimental conditions requires the application of a multivariate data analysis to maximize the extraction of information. In this point is where metabolomics and chemometrics converge in the field of FF. Chemometrics utilizes different multivariate data analysis methods and principles to evaluate all experimental variables at the same time and analyse them from a chemical, mathematical, and statistical point of view. This tool allows designing optimal protocol conditions which provides objective data evaluation and leads to the extraction of meaningful information. Data can be quantitatively modelled and visually presented. The most common analysis approaches used in chemometrics are exploratory (it detects patterns, tendencies, or clusters), classification, and discriminant (samples are classified in categories) and regression and prediction models (applied for reinforcing a sought data relationship) [133,134].

Metabolomics and chemometrics can be applied to both spectroscopic and chromatographic data, as well as targeted and non-targeted methods that are employed in the identification of FF or the specific origin of a product. In fact, they have been applied for the analysis of complex spectroscopic and chromatographic data pools in order to authenticate origin or determine fraud in edible oils, having special importance in the case of EVOO for its economical repercussion [11,134].

For instance, the geographical origin of Arbequina variety EVOOs was determined by developing chromatographic (both HPLC and GC) fingerprints. Geographical origin was explored by analysing data through the exploratory technique (principal component analysis, PCA) and two classification methods (soft independent modelling of class analogy, SIMCA, and partial least square-discriminant analysis, PLS-DA) [102]. Another work using chromatographic methods coupled to chemometrics that allow the analysis of the metabolic profile of EVOO has been also demonstrated to be capable of discriminating samples by geographical origin. The metabolites with highest discrimination potential, obtained by a supervised multivariate PLS model, were phenols and sterols. In the group of the phenolic compounds were identified some molecules capable of discriminate the geographical origin, among them some anthocyanins (cyanidin 3-O-xylosyl-rutinoside), isoflavonoids (6”-O-acetylglycitin) and phenolic acids belonging to the hydroxycinnamic class (p-coumaroyl glucose or p-coumaric acid). In the family of the sterols cholesterol, spirostanols, ergosterols, steryl esters and stigmasterol, furostanol and cycloartanol derivatives were underlined [131]. For fraud evaluation, HPLC-UV was used for obtaining fingerprinting of Arbequina variety EVOOs. Contaminations with Picual variety, refined olive oil, and sunflower oil were identified when coupling chromatographic results to PLS regression [135]. In another study, the application of SIMCA to results obtained with selected ion flow tube MS could differentiate samples based on their volatile profile Whereas PLS approach allow the identification different kinds of EVOO adulterations based on several main target compounds [136]. Therefore, in both chromatographic based works the best approach to determine adulteration type or geographical origin was achieved when coupling chromatographic data to PLS model [102,135,136].

Spectroscopic data obtained with RAMAN has been repeatedly used for assessing authenticity of fatty products and edible oils through different modeling methods such as PCA or PLS, among others [137,138]. Apart from RAMAN, NIR technique in combination with PLS was also demonstrated as a good prediction model for identifying and quantifying the adulteration of the olive oil with soybean, sunflower, corn, or canola oil [16,139]. While NIR data analyzed with PCA or SIMCA approaches are permitted to distinguish between sample classes [139].

### 4.4. Hyperspectral Imaging and Chemometrics

Hyperspectral imaging (HSI) or image spectroscopy provides both spectral and spatial information of an analyzed item. The spatial feature improves the authentication of complex and heterogeneous samples, whereas the spectral information permits to identify a wide range of multi-constituent surface and subsurface features [140]. HSI data can be obtained through electromagnetic measurements, NIR, MIR, or Raman spectroscopy, or through confocal laser microscopy scanners, X-ray spectroscopy or 3D ultrasound imaging, among others [11]. This technique presents many advantages since it is non-destructive, requires minimal amount of sample, has a low rate of reagents consumption and provides rapid results [141]. Consequently, HSI is time- and cost-efficient since experimental times are reduced, minimize the reagent cost and avoid those related with waste treatment [142]. However, this technique can also provide complex matrixes of data thus it is frequently coupled to chemometrics analysis. For its rapidness, this technique has been also utilized for the analysis of vegetal oils, mostly as a tool for evaluating quality parameters [143]. Quality parameters of VOO samples like acidity, peroxide value, and moisture content were determined in samples collected along different seasons. Samples were analyzed in the region of 900–1700 nm and results analyzed through PLS regressions and then compared against those from other analytical methods. Acidity, moisture, and peroxide values obtained using HSI coupled to PLS were comparable to analytical results [111]. This technique was also applied for differentiating three kinds of blends that were assayed as unflavored and flavored with three aromatic compositions. Samples were analyzed in the region of 400 to 1000 nm being of special relevance the region ranging from 400 to 570 nm and around 695 nm for detecting differences among samples [106]. The application of this technique, NIR-HSI, has been applied to other vegetal oils such as that extracted from sesame seeds. Varieties of sesame oil were identified by this non-destructive assay. After recording data from the spectral region of 874–1734 nm, different identification models were established by utilizing several algorithms. The most relevant spectral segment for performing the discrimination of varieties was found between 921 and 1663 nm [108]. VIS-NIR-HIS was also applied for the on-line assessment of the quality of frying oil during the heating process. The quality parameter studied included free fatty acid value, viscosity, and total polar compounds. A portable HIS was used for collecting spectral data in the wavelength range of 350–2500 nm. Extreme values contained too much noise and were refused. From a narrower spectral range, from 400 to 1750 nm, 36 spectra were randomly selected for developing PLS calibration models. Comparison among observed values and predicted ones displayed R^2^ higher than 0.9 for all parameters, being especially accurate for predicting the acid value (R^2^ of 0.95) and total polar compounds (R^2^ of 0.98) [110].

Therefore, the application of the abovementioned spectral and/or chromatographic techniques for collecting data and coupled to a multivariate data analysis model can provide accurate tools for identifying samples. They provide a fingerprint, metabolomic or HIS-based, to specific samples that permits to discriminate them from others with fraudulent compositions or from different geographical areas.

## 5. Conclusions

The analytical chemistry of foods has evolved significantly in recent decades, which has made it possible to have a greater knowledge about the composition of foods and the changes that they undergo due to time and/or processing. Yet, despite all these advances, cases of fraud still occur. In this sense, olive oil is one of the foods most susceptible to food fraud, both for adulteration or falsification of its origin/variety. To avoid this situation, numerous techniques have been developed such as FTIR spectroscopy, Raman spectroscopy, U–Vis spectroscopy, GC, HPLC, or DNA analysis, which have been used for the detection and quantification of adulterants and confirm the geographical region or the variety used to obtain the olive oil. However, all the mentioned techniques present drawbacks. The chromatographic methods detect the FF based on some marker compounds, so the information obtained is easy to evaluate. However, these techniques are time-consuming, and they involve a complex sample preparation and the use of toxic solvents. On the other hand, spectroscopic techniques do not need sample preparation or toxic solvents, but large amounts of data are generated, whose interpretation is laborious. In this sense, some authors have proposed that spectroscopic techniques could be useful to upgrade the chromatographic techniques. In the case of other methodologies, such as DNA and protein-based methods or metabolomic approach, although they have been demonstrated to be efficient techniques to detect adulterations and authenticate the origin/cultivar of olive oil samples, their use is still limited and further improvements are necessary.

## Figures and Tables

**Table 1:** Tests carried out with spectrometric techniques to determine and/or quantify adulterations in olive oil.

Technique	Adulterant	Detection (%)	Quantification (%)	Conditions	Ref.
NIR	Soybean Oil	-	1.76	12,000–4000 cm^−1^	[16]
NIR	Olive Pomace Oil	-	3.27	8000–2000 cm^−1^	[17]
NIR	Corn, sunflower, soya, walnut and hazelnut oil	2	0.57, 1.32, 0.96, 0.56, 0.57	12,000–4000 cm^−1^	[18]
NIR	Sunflower Oil	1	-	2498–1100 nm	[19]
NIR	Adulterants	1		5280 cm^−1^	[20]
FT-IR	Low-cost edible oils	5	-	4000–500 cm^−1^	[21]
FT-IR	Olive Pomace Oil	-	3.28	4000–400 cm^−1^	[17]
FT-IR	Peanut Oil	1	-	3050–600 cm^−1^	[22]
FT-IR	Peanut Oil	5	-	4000–400 cm^−1^	[23]
FT-IR	Hazelnut Oil	25	-	3100–800 cm^−1^	[24]
FT-IR	Palm, corn, canola and sunflower oil	-	1	1500–1000 cm^−1^	[25]
FT-IR	Soybean and tea seed oil		1	4000–650 cm^−1^	[26]
MIR	Old olive oil		1–50	4000–600 cm^−1^	[27]
MIR	Soybean Oil		4.89	4000–350 cm^−1^	[16]
MIR	Corn-sunflower mixture, cottonseed, and rapeseed	5		4000–650 cm^−1^	[28]
Raman	Soybean Oil		1.57	3500–50 cm^−1^	[16]
Raman	Olive Pomace Oil		1.72	3700–400 cm^−1^	[17]
Raman	-		5	2400–250 cm^−1^	[29]
Raman	Sunflower oil		1	3100–560 cm^−1^	[30]
Raman	Soybean oil	1		1800–1000 cm^−1^	[31]
Raman	Waste cooking oil		2.5	3500–100 cm^−1^	[32]
UV-VIS	Refined oil	<10		0–650 nm	[33]
UV-VIS	Frying oils	1		0–650 nm	[34]
UV-VIS	Olive oil	8.4		60–700 nm	[35]
NMR	Lampante olive oil, Refined olive oil	5		-	[36]
NMR	Seed and nut oils	10		-	[37]

**Table 2:** Tests carried out with different identification and quantification techniques to determine and/or quantify adulterations in olive oil.

Technique	Adulterant	Conditions	Ref.
GC	Soybean, corn, sunflower oil	Column Agilent CP-Sil88 (50 m × 0.25 mm, 0.20 μm). FID (HP 6890N, Agilent, 250 °C). t_0_ = 165 °C, 25 min; gradient of 5 °C/min t_f_ = 195 °C.Percentage of adulteration detection: 1–3%	[101]
HPLC	Hazelnut, olive and their mixtures	Column Spherisorb ODS2 (octadodecylsilane) (46 cm × 0.25 m, 5 µm). 25 °C, 50 min. (A): A–Act (64:36, *v/v*) 1 mL/min. Percentage of adulteration detection: 2%	[104]
HPLC	Hazelnut	Kromasil 100-5C18 (3.2 mm × 250 mm; 5 μm). (A): W/AA (97:3, *v/v*). (B): M/Act (50:50, *v/v*). 30 °C, 0.490 mL/min. PDA-100, 280 nm. Isocratic (95% A–5% B, 15 min), gradient (100% B, 25 min) back to 5% B, 20 min. Percentage of adulteration detection: 5%	[105]
HSI	Olive oil	400–570 nm. Competitive adaptive reweighted sampling (CARS), successive projections algorithm (SPA), and x-loading weights (x-LW)	[106]
HSI	Sesame oil	325–1075 nm. Support Vector Machine-Multiclass Forward Feature Selection (SVM-MFFS)	[107]
HSI	Sesame oil	874–1734 nm. Least Squares-Support Vector Machine (LS-SVM) and the Linear Discriminant Analysis (LDA)	[108]
HSI	Edible and waste cooking oils	350–2500 nm. Unweighted Distance Method and Interior Square Sum Distance	[109]
HSI	Frying oils	400–1750 nm. PLS calibration models	[110]
HSI	Virgin olive oils	900–1700 nm. Genetic Algorithm (GA), Least Absolute Shrinkage and Selection Operator, and Successive Projection Algorithm (SPA)	[111]
IRMS	Glycerol, fatty alcohols	δ13C, δ18O	[74]
IRMS	Palmitic acid, palmitoleic acid, stearic acid, oleic acid, linoleic acid, linolenic acid	δ13C, bulk. Vegetable oils can be classified using the isotopic ratios of the bulk oil, the fatty acids, and also the composition of the fatty acids	[73]
IRMS	Phytol, geranyl geraniol, citrostadienol, docosanol, tetracosanol, hexacosanol	δ13C. Percentage of adulteration detection: 3%	[78]
IRMS	Methyl palmitoleate, methyl palmitate, methyl oleate	δ13C. Use of 3 FAME peaks enabled greater differentiation between samples of different geographic origin compared to using the isotopic ratios of the bulk oils	[75]
Enzymes	Hazelnut proteins	Indirect competitive ELISA and direct immunosensor. For biosensor, LOD 0.08 μg/g olive oil, assay time 4.5 min	[112]
Enzymes	Aflatoxin B1	Immunostrips and indirect competitive ELISA. For strips, visual LOD 1 ng/mL, assay time 15 min	[113]
Enzymes	OrganophosporusPesticides	Indirect and direct fluorescent competitive immunosensor. High sensitivity of the fluorescence transducer	[114]

Solvents: W: Water; AA: Acetic Acid; M: Methanol; Act: Acetonitrile; A: Acetone. Columns (Length × Internal Diameter, particle size).

## Data Availability

Data sharing is not applicable to this article.
